# A phage-derived promoter *SD13* enables strong and cross-host gene expression in *Streptomyces* and *Escherichia coli*

**DOI:** 10.1007/s00253-026-13981-0

**Published:** 2026-08-04

**Authors:** Nana Lu

**Affiliations:** School of Marine and Biological Engineering, Yancheng Teachers University, Yancheng, 224002 China

**Keywords:** *Streptomyces* bacteriophage, *SD13* promoter, Cross-host expression, Strong promoter, Gene expression regulation

## Abstract

**Abstract:**

Efficient and tunable promoters are essential tools for metabolic engineering and synthetic biology in *Streptomyces*. In this study, the lytic bacteriophage phiSASD1 was exploited as a source of novel regulatory elements. Fifteen candidate promoter fragments were identified through bioinformatic prediction and systematically evaluated using a catechol 2,3-dioxygenase (*xylE*) reporter system in *Streptomyces lividans* TK54 and *Escherichia coli* JM109. Among these candidates, seven fragments exhibited measurable promoter activity, with P*SD13* showing the highest transcriptional strength. In *S. lividans*, P*SD13* displayed up to a 9.79**-**fold higher activity than the widely used strong promoter PermE*, while retaining detectable activity in *E. coli*, indicating excellent cross-host compatibility. Sequence analysis combined with 5′ RACE revealed that P*SD13* possesses a typical σ⁷⁰**-**dependent promoter architecture, with its core functional region located between − 70 to + 9 bp relative to the transcription start site. Furthermore, P*SD13* efficiently drove the soluble expression of phage endolysin in *E. coli*, potentially reducing inclusion body formation compared with conventional T7-based expression systems. Collectively, these results identify P*SD13* as a phage-derived promoter exhibiting strong activity in both *Streptomyces* and *E. coli*, suggesting its potential as a useful genetic element for *Streptomyces* engineering and heterologous gene expression.

*****Key points***:**

• *Strong transcriptional promoters were systematically identified from Streptomyces phages.*

• The novel SD13 promoter shows higher activity than PermE* and works across hosts.

• *SD13 promoter efficiently expresses soluble heterologous proteins, advancing Streptomyces synthetic biology.*

**Supplementary Information:**

The online version contains supplementary material available at https://doi.org/10.1007/s00253-026-13981-0.

## Introduction

Synthetic biology increasingly relies on the coordinated use of multiple microbial chassis to accelerate the design–build–test–learn (DBTL) cycle for natural product discovery and metabolic engineering. Among available microbial hosts, *Escherichia coli* and Streptomyces have emerged as two complementary platforms with distinct yet highly synergistic advantages. *E. coli* serves as an ideal rapid-prototyping host owing to its fast growth, well-developed genetic toolbox, and high transformation efficiency, allowing efficient construction and preliminary evaluation of genetic circuits (Zhang et al. 2022a, b). In contrast, *Streptomyces* species possess exceptional metabolic capacities, abundant precursor supplies, and sophisticated secondary metabolic networks, making them indispensable industrial chassis for the biosynthesis of antibiotics, anticancer agents, enzymes, and other high-value natural products (Javorova et al. 2024). Consequently, modern metabolic engineering frequently requires iterative engineering between these two hosts, where regulatory elements developed in one organism are expected to function efficiently in the other. Such cross-host compatibility has become an important prerequisite for standardized synthetic biology and modular pathway engineering.

Despite these complementary advantages, the construction of universal genetic expression systems remains a major challenge because *E. coli* and *Streptomyces* possess fundamentally different transcriptional architectures. As representatives of Gram-negative and Gram-positive bacteria, respectively, they differ substantially in genome GC content, promoter sequence composition, sigma factor repertoires, RNA polymerase (RNAP) recognition mechanisms, transcription factor networks, and chromosomal organization (Zhang et al. 2025; Shi et al. 2024). These intrinsic biological differences generate pronounced genetic expression barriers that frequently lead to dramatic reductions in promoter activity when regulatory elements are transferred between hosts. Even promoters exhibiting strong activity in one microorganism often become substantially weakened or completely inactive in another, severely restricting the portability of synthetic biological components and increasing the complexity of pathway optimization (Keating and Young 2023).

Promoters represent the central cis-regulatory elements controlling transcription initiation and therefore largely determine the efficiency, stability, and predictability of heterologous gene expression. During the past decade, considerable efforts have been devoted to identifying constitutive promoters, engineering synthetic promoters, and constructing promoter libraries in *Streptomyces* (Zhao et al. 2024; Li et al. 2023; Stegmüller et al. 2024). Several endogenous promoters, including PermE*, P*kasO*, P*hrdB*, P*SP44*, and P*stnY*, have significantly expanded the genetic toolbox available for metabolic engineering and activation of silent biosynthetic gene clusters (Guo et al. 2023; Huang et al. 2025). Nevertheless, these promoters were primarily optimized for *Streptomyces* hosts and generally exhibit poor transcriptional performance or complete inactivity in *E. coli*. Likewise, many widely used *E. coli* promoters, such as T7 and *lac*-derived systems, rely on host-specific transcriptional machinery and are unsuitable for direct application in high-GC actinomycetes (Baltz 2024; Nah et al. 2017). Therefore, current promoter resources remain largely host-dependent and cannot satisfy the increasing demand for standardized cross-host genetic regulation.

The lack of efficient cross-host promoters has become one of the major obstacles limiting microbial chassis interoperability. Existing cross-host regulatory elements often suffer from several shortcomings. First, promoter strength is frequently unbalanced between different hosts, resulting in inconsistent transcriptional outputs during pathway transfer (Ali et al. 2024). Second, promoter performance is strongly influenced by host-specific RNAP recognition and surrounding genomic context, limiting their adaptability across phylogenetically distant microorganisms (Yang et al. 2024). Third, excessively strong expression systems, particularly bacteriophage T7-derived systems, often impose substantial metabolic burdens and promote inclusion-body formation during heterologous protein production (Liu et al. 2021). These limitations reduce the reusability of genetic components and hinder the realization of truly more modular synthetic biology platforms. Therefore, identifying promoters capable of maintaining robust and balanced transcription across distinct microbial hosts has become an important objective in contemporary promoter engineering.

Bacteriophages provide a particularly attractive resource for discovering such regulatory elements. During infection, bacteriophages must rapidly hijack the host transcriptional machinery to complete efficient genome replication and progeny production. Consequently, phage genomes have evolved highly efficient promoters that can be rapidly recognized by host RNA polymerases (Kronheim et al. 2023). Unlike many endogenous bacterial promoters that evolved under complex physiological regulation, phage promoters are often optimized for maximal transcriptional efficiency while retaining relatively simple regulatory architectures. Previous investigations of *Streptomyces* phages, including φC31 and φBT1, have demonstrated that phage-derived promoters can exhibit remarkably strong transcriptional activities and have been successfully incorporated into various genetic engineering systems (Howe and Smith 1996; Gregory et al. 2003; Li et al. 2020). Nevertheless, systematic exploration of phage-derived promoters remains limited, particularly regarding promoters possessing broad host compatibility. Most previous studies focused primarily on improving promoter strength within a single host rather than helping to reduce transcriptional barriers between different microbial chassis (Zhang et al. 2022a, b). As a result, whether phage genomes harbor naturally evolved promoters with activity in both *Streptomyces* and *E. coli* remains largely unexplored.

The lytic bacteriophage phiSASD1 infects *Streptomyces avermitilis*, an important industrial producer of avermectins, and its genome has been completely sequenced (Wang et al. 2010). Because productive phage infection requires rapid activation of viral transcription immediately after host invasion, the phiSASD1 genome is expected to contain highly efficient native promoters. However, these regulatory elements have not been systematically characterized, and their transcriptional properties, structural features, and host adaptability remain unknown. This knowledge gap limits the exploitation of phiSASD1-derived regulatory elements in synthetic biology and metabolic engineering.

In the present study, we systematically mined promoter candidates from the phiSASD1 genome through a combination of bioinformatic prediction and experimental validation. Candidate promoters were quantitatively evaluated in both *Streptomyces lividans* and *E. coli* using a unified reporter system to assess transcriptional strength and host compatibility. The strongest promoter, P*SD13*, was further characterized through transcription start site determination, conserved motif analysis, mutational studies, and promoter truncation experiments to elucidate its functional architecture. Importantly, P*SD13* exhibited not only higher transcriptional activity than the widely used PermE* promoter in *Streptomyces*, but also retained detectable activity in *E. coli*, enabling efficient soluble heterologous protein expression across hosts. These findings suggest that P*SD13* is a phage-derived promoter with cross-host activity and the potential to alleviate host-dependent transcriptional constraints, providing a useful regulatory element for microbial chassis engineering and synthetic biology applications.

## Materials and methods

### Strains, bacteriophage, and plasmids

*Streptomyces avermitilis* ATCC31267, used as the host strain for bacteriophage phiSASD1, was maintained in our laboratory. Bacteriophage phiSASD1 is a lytic *Streptomyces* phage previously isolated, and its genome has been sequenced and annotated (GenBank accession number: GQ379227). *E. coli* JM109 was used for plasmid construction, propagation, promoter activity assays, and heterologous protein expression. *Streptomyces lividans* TK54 was employed as the *Streptomyces* expression host. Detailed genotypes and descriptions of all bacterial strains are provided in Supplementary Table S1.

For promoter characterization, the *E. coli*–*Streptomyces* shuttle vector pSET152 and the reporter plasmid pIJ4083 carrying the *xylE* gene were used to construct the promoter probe vector pSX152. The *xylE* gene encodes catechol 2,3–dioxygenase (C23O), whose activity can be quantified using a catechol-based colorimetric assay. The constitutive promoter PermE*, derived from *Saccharopolyspora erythraea*, was used as a positive control, while the empty vector pSX152 served as a negative control. Detailed information on all plasmids, including plasmid backbones, selectable markers, inserted genetic elements, and their applications, is summarized in Supplementary Table S2.

### Culture conditions

*E. coli* strains were cultured in LB (Luria–Bertani) liquid medium supplemented with appropriate antibiotics at 37 °C with shaking at 200 rpm. *S. avermitilis* and *S. lividans* TK54 were routinely maintained on YMS (Yeast extract, Maltose and Starch medium) agar plates and cultured in YEME (Yeast Extract–Malt Extract) liquid medium. For liquid cultivation, strains were incubated at 28 °C with shaking at 200 rpm, while solid cultures were grown on agar plates containing 2% (w/v) agar. Bacteriophage phiSASD1 was propagated using the double-layer agar method, and high-titer phage lysates were prepared under optimal host growth conditions.

### Extraction of bacteriophage genomic DNA

High-titer phage lysates were first treated with DNase I and RNase A to remove residual host nucleic acids. Subsequently, SDS and proteinase K were added to disrupt the phage capsid and release genomic DNA. Proteins were removed by phenol/chloroform extraction, followed by ethanol precipitation to recover the DNA. The integrity of the extracted DNA was verified by 1% agarose gel electrophoresis, and DNA concentration and purity were determined using a NanoDrop spectrophotometer.

### Bioinformatic analysis of promoter sequences

Putative promoter sequences from the phiSASD1 genome were identified using a combined computational pipeline. Intergenic regions upstream of annotated ORFs were extracted based on GenBank annotation (accession number: GQ379227) and used as input sequences (Wang et al. 2010). Promoter prediction was performed using BDGP Neural Network Promoter Prediction (http://www.fruitfly.org/seq_tools/promoter.html) (Reese 2001), BPROM (Softberry; http://www.softberry.com) (Solovyev and Salamov 2011). A minimum score threshold of 0.80 was applied in BDGP to reduce false-positive predictions, while default parameters were used for BPROM. Transcription factor binding site prediction was performed using PRODORIC database (http://www.prodoric.de; accessed 2024) (Münch et al. 2003) and BPROM.

### Construction of promoter probe plasmids

Candidate promoter fragments were amplified from phiSASD1 genomic DNA using gene-specific primers and a high-fidelity DNA polymerase. PCR products were verified by agarose gel electrophoresis, purified, digested with the appropriate restriction enzymes, and ligated into the promoterless *xylE* reporter vector pSX152 to generate promoter-reporter fusion constructs. The ligation mixtures were transformed into* E.* JM109 competent cells, and positive transformants were selected on antibiotic-containing media. Recombinant plasmids were verified by colony PCR and DNA sequencing to confirm the correct insertion of promoter fragments. The verified constructs were subsequently introduced into *E. coli* JM109 only for promoter activity assays. All primers used in this study, together with their sequences and corresponding applications, are listed in Supplementary Table S3. The complete nucleotide sequences of all promoter fragments, including P*SD13* and the other experimentally validated active promoter candidates, are provided in Supplementary Table S4.

### Construction of recombinant strains

Verified pSX152-derived promoter-reporter plasmids were first introduced into *E. coli* ET12567/pUZ8002 and then transferred into *S. lividans* TK54 by intergeneric conjugation. Because pSX152 was constructed on the pSET152 integrative vector backbone, the promoter-*xylE* expression cassettes were integrated into the *S. lividans* chromosome through φC31 integrase-mediated *attP*/*attB* site-specific recombination, rather than maintained as autonomously replicating plasmids. Exconjugants were selected on media containing appropriate antibiotics and verified by colony PCR. In parallel, recombinant plasmids carrying different promoter fragments were transformed into *E. coli* JM109 and maintained as plasmid-borne constructs for promoter activity assays in *E. coli*.

### Promoter activity assay

For promoter activity assays in *E. coli*, recombinant *E. coli* JM109 strains carrying the corresponding pSX152-derived promoter-*xylE* reporter plasmids were cultured overnight in LB medium supplemented with the appropriate antibiotic at 37 °C with shaking. After 12 h of cultivation, cells were collected by centrifugation, resuspended in pre-cooled sample buffer, and disrupted by sonication. The cell lysates were centrifuged, and the resulting supernatants were used as crude enzyme extracts for C23O activity determination.

For promoter activity assays in *Streptomyces lividans* TK54, verified recombinant strains were first inoculated into YEME medium and cultured at 28 °C with shaking at 200 rpm for 2 days to prepare seed cultures. The seed cultures were then inoculated at 1% (*v/v*) into 50 mL YEME fermentation medium, with three independent flasks prepared for each strain. Cultures were incubated at 28 °C with shaking at 250 rpm, and samples were collected at 12, 36, and 60 h.

For each time point, 40 mL of culture was collected. Of this, 10 mL was used to determine cell dry weight, and 30 mL was used for *xylE* activity measurement. For dry weight determination, cells were harvested by centrifugation, washed twice with sample buffer, and dried at 80 °C to constant weight. For C23O activity measurement, cells from 30 mL culture were harvested by centrifugation at 12,000 rpm for 10 min, washed twice with sample buffer, and resuspended in 10 mL of pre-cooled sample buffer. Cells were disrupted by sonication on ice for 8 cycles of 15 s, with 30 s intervals between cycles. Triton X-100 was then added to a final concentration of 0.1%, and the mixture was incubated on ice for 15 min. After centrifugation at 12,000 rpm for 5 min, the supernatant was collected as crude enzyme extract.

For the enzyme reaction, 200 μL of crude enzyme extract was added to 4 mL of assay buffer pre-warmed at 30 °C. The reaction was carried out at 30 °C for 20 min, and the absorbance was measured at 375 nm. C23O activity was calculated as:

$$\mathrm{m}\mathrm{U}\ (\mathrm{n}\mathrm{m}\mathrm{o}\mathrm{l}\cdot \mathrm{m}\mathrm{i}\mathrm{n}-1)\hspace{0.17em}=\hspace{0.17em}30.03\hspace{0.17em}\times \hspace{0.17em}\Delta \mathrm{A}375 / \mathrm{t}\mathrm{i}\mathrm{m}\mathrm{e}$$

One milliunit (mU) was defined as the amount of enzyme required to produce 1 nmol of 2–hydroxymuconic semialdehyde per minute at 30 °C. Promoter activity was normalized to cell dry weight and expressed as mU/mg. For *S. lividans* TK54, promoter activities were measured using single-copy chromosomally integrated pSX152-derived cassettes, whereas for *E. coli* JM109, promoter activities were measured using plasmid-borne pSX152-derived constructs. Thus, the *Streptomyces* assays reflected a fixed chromosomal integration context, while the *E. coli* assays reflected a plasmid-based reporter context. The PermE* promoter and empty pSX152 vector were used as positive and negative controls, respectively. All experiments were performed with three independent biological replicates.

### Determination of transcription start site (TSS)

The transcription start site of the P*SD13* was determined using Rapid Amplification of cDNA Ends (5′ RACE). Total RNA was extracted from *S. avermitilis* at different time points following phage infection and reverse-transcribed into cDNA. Nested PCR was performed using ORF13-specific primers to amplify the transcription start region. PCR products were sequenced, and the TSS was identified based on the anchor sequence and the first nucleotide immediately downstream of the poly(A) tail.

### Identification of functional promoter regions

To clarify the rationale for P*SD13* truncation design, multiple bioinformatic and experimental analyses were performed as follows: The truncation strategy of P*SD13* was guided by multiple bioinformatic and experimental evidences. First, the transcription start site was determined by 5′ RACE, which served as a positional reference for downstream boundary design. Second, promoter prediction was performed using BDGP (score threshold > 0.8), and BPROM to identify putative −10 and −35 elements. Third, σ^70^ consensus motif analysis was conducted using the PRODORIC database and BPROM to confirm canonical promoter architecture. Based on these analyses, stepwise 5′- and 3′-end deletions were designed to progressively remove upstream regulatory regions while retaining the predicted core promoter region, thereby enabling functional identification of the minimal promoter element.

### Heterologous expression of endolysin driven by the PSD13

To evaluate the protein expression capability of the phage-derived P*SD13*, the endolysin gene from bacteriophage phiSASD1 was amplified by PCR using gene-specific primers (endolysin- F: 5′-CGTCTAGAATGGACAGCCCTTCGCGT-3′; endolysin- R: 5′-AGGATCCTCAGGCGGTCACGGTCC-3′) and cloned downstream of the P*SD13* in the expression vector pIJ13, generating the recombinant plasmid pIJ13-lysin. The construct was verified by DNA sequencing and transformed into *E. coli* JM109 for protein expression. The empty vector pIJ13 was transformed into *E. coli* JM109 and used as a negative control.

Recombinant strains were cultivated in LB medium supplemented with the appropriate antibiotic at 37 °C with shaking at 200 rpm. Cells were harvested after 8 h and 10 h of cultivation by centrifugation and resuspended in lysis buffer. Cell disruption was performed by sonication on ice, followed by centrifugation to separate soluble and insoluble protein fractions. Protein samples from the total lysate, supernatant, and pellet fractions were mixed with SDS-PAGE loading buffer, boiled for 10 min, and analyzed by 12% SDS-PAGE. Protein bands were visualized by Coomassie Brilliant Blue staining to evaluate the expression level and solubility of the recombinant endolysin.

### Data processing and statistical analysis

All experimental data were presented as mean ± standard deviation (mean ± SD). Statistical differences among groups were evaluated using one-way analysis of variance (ANOVA), and differences were considered statistically significant at *P* < 0.05. All statistical analyses and data visualization were performed using GraphPad Prism 5.0 software.

## Results

### Identification of putative promoters in phage phiSASD1

Based on the complete genome sequence of the *S. avermitilis* bacteriophage phiSASD1, a total of 43 open reading frames (ORFs) were predicted. Intergenic regions between adjacent ORFs were screened for potential promoter sequences and further analyzed using the BDGP promoter prediction program. With the minimum promoter score threshold set at 0.8, nine high-scoring candidate promoter regions were identified, with prediction scores ranging from 0.82 to 0.97 (Table 1). Based on ORF annotation from the NCBI GenBank database, upstream intergenic sequences were further selected as candidate promoter fragments. In total, 15 putative promoter regions associated with ORF1, ORF3, ORF5, ORF8, ORF10, ORF11, ORF12, ORF13, ORF15, ORF17, ORF18, ORF19, ORF21, ORF38, and ORF40 were obtained for experimental validation (Table 2). These candidate fragments ranged from 92 to 703 bp in length and were associated with genes encoding proteins such as Cas4 exonuclease, DNA polymerase I, RNA polymerase subunit alpha, holin, and endolysin, suggesting potential roles in phage replication and lysis processes.

**Table 1:** High-scoring promoter regions predicted in the phiSASD1 genome

Number	Start (bp)	Stop (bp)	Score	Promoter sequence^a^ (5′ → 3′)
1	4117	4162	0.87	GTTGTGGAACTACCCGCGTGCTCCCGCT GCATCATCCTCG**C**CGGCCGCGA
2	12,067	12,112	0.94	GCCAGTTGGATGGTGCAGGTCTACAACG GTACCGTTATAT**T**CCGCTACAC
3	18,885	18,930	0.97	TATTTTTGTTTCGTCCCAGTAATAGAAAA GATAGTAACGC**T**CCTCGCCTT
4	19,636	19,681	0.88	CAGTTGGCCGCCTACCGGTACGCGGACG TGATCATTGACC**C**GGAGGGCAA
5	25,381	25,426	0.84	CTGATGGAAAACCAGGACTTCCCTCAGG ACATCTTTCCGC**T**GAACTACAC
6	30,227	30,272	0.92	CTCACTTGACCCGACTCAATTGAGTCGC CTACTATGACAA**C**GCGTCCACA
7	32,201	32,246	0.82	ACGGCTTGACTCGACTCAGTTGAGTCGG CTACTTTCGGCA**T**TCCAGCCGC
8	32,442	32,487	0.86	ACCCTTGACCTGTTCATCGTGGCCGGCG AGATCATGATGC**T**GGTGGCCGC
9	35,074	35,119	0.94	GCGGCTTGACTCGACTCAATTGAGCCGG CTAAATTGGAGC**C**CTCGCCAGG

^a^Bold letters indicate the transcription start site (TSS)

**Table 2:** Lengths of putative promoter fragments and predicted proteins encoded by the corresponding ORFs

Potential promoter	Length (bp)	Putative product^a^
ORF1	561	Cas4 family exonuclease
ORF3	199	PAPS _reductase
ORF5	142	–
ORF8	703	–
ORF10	258	–
ORF11	106	primase/helicase
ORF12	187	DNA polymerase I
ORF13	265	DNA-directed RNA polymerase subunit alpha
ORF15	150	–
ORF17	660	HNH endonuclease
ORF18	176	–
ORF19	687	holin
ORF21	188	Terminase small subunit
ORF38	92	–
ORF40	144	endolysin

^a^The symbol “–” indicates that no homologous function was identified

### Promoter activity of candidate fragments in S. lividans TK54

**Fig. 1:**
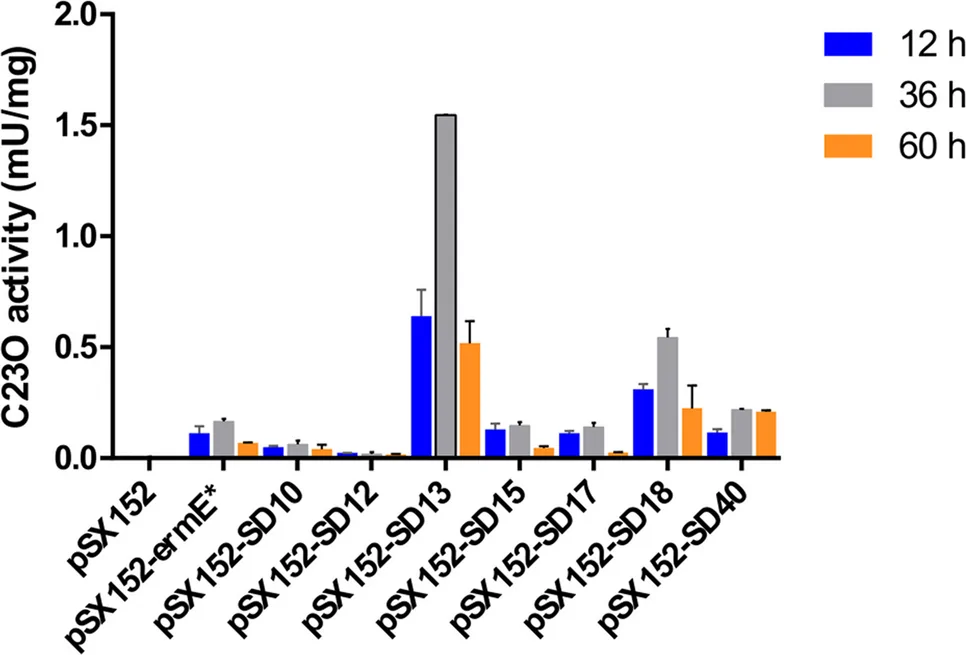
Screening and comparative analysis of promoter activities of phiSASD1-derived promoter fragments in *Streptomyces lividans* TK54. Error bars represent the standard deviation of the mean from three independent experiments

To experimentally evaluate promoter activity, the 15 candidate fragments were individually cloned into the promoter probe vector pSX152 and integrated into the chromosome of *S. lividans* TK54. Promoter strength was assessed by measuring the activity of the *xylE* reporter gene. The constitutive promoter PermE* was used as a positive control, while the empty vector served as a negative control. Among the 15 candidates, seven promoters (P*SD10*, P*SD12*, P*SD13*, P*SD15*, P*SD17*, PS*D18*, and P*SD40*) exhibited detectable transcriptional activity (Fig. 1). Notably, P*SD13* showed the highest activity, reaching 6.72-, 9.79-, and 5.06-fold that of PermE* at 12 h, 36 h, and 60 h, respectively (Fig. 1). P*SD18* also exhibited strong activity, approximately 3.38-fold higher than PermE*, while P*SD15* displayed a comparable level of activity to PermE* (Fig. 1). Time-course analysis revealed that all active promoters reached peak C23O activity at 36 h, followed by a decline at 60 h. P*SD40* maintained relatively high activity at 60 h (Fig. 1). Interestingly, some fragments with high prediction scores (e.g., SD1 and SD38) showed no detectable promoter activity (data not shown), whereas P*SD15*, despite a relatively low prediction score, exhibited substantial transcriptional strength (Fig. 1).

### Expression activity of promoter fragments in E. coli JM109

**Fig. 2:**
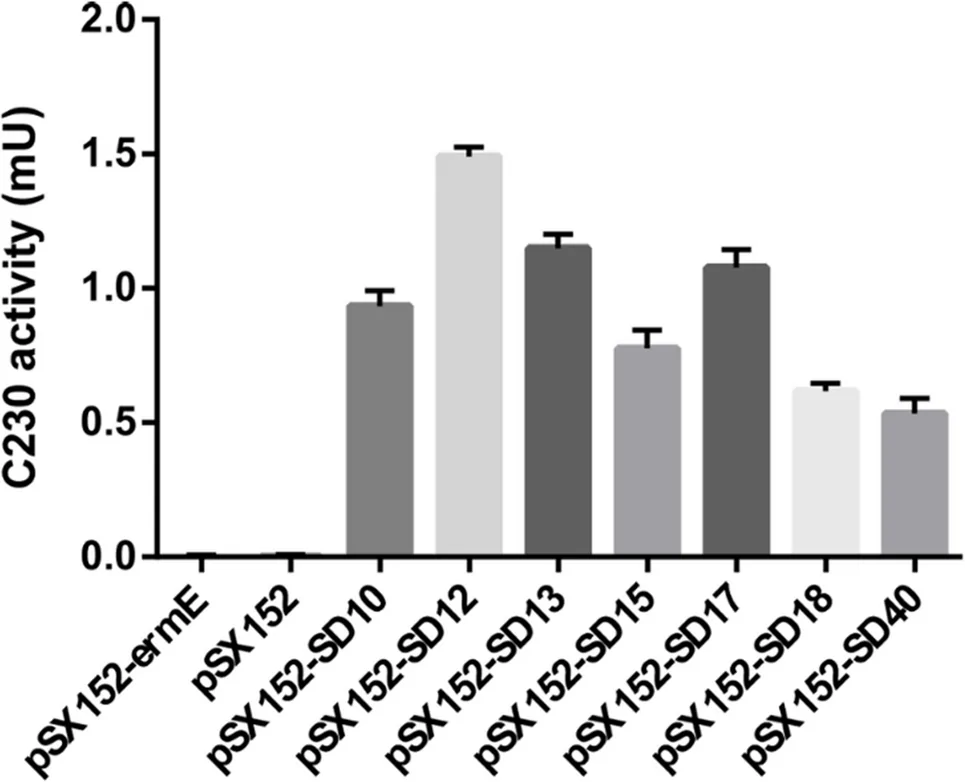
Expression activity of bacteriophage phiSASD1 promoter fragments in *E. coli* JM109

To assess host compatibility, the seven active promoter fragments identified in *S. lividans* were further evaluated in *E. coli* JM109 using the same reporter system. The results showed that all seven promoters retained transcriptional activity in *E. coli* (Fig. 2), whereas the positive control promoter PermE* exhibited no detectable activity under the same conditions. Notably, P*SD13* maintained relatively high expression levels, suggesting its cross-host activity. In addition, several promoters (e.g., P*SD10*, P*SD12*, and P*SD17*) that showed relatively low activity in *S. lividans* exhibited enhanced activity in *E. coli*, highlighting the influence of host-specific transcriptional machinery on promoter performance (Fig. 2).

### Expression of endolysin driven by the PSD13

**Fig. 3:**
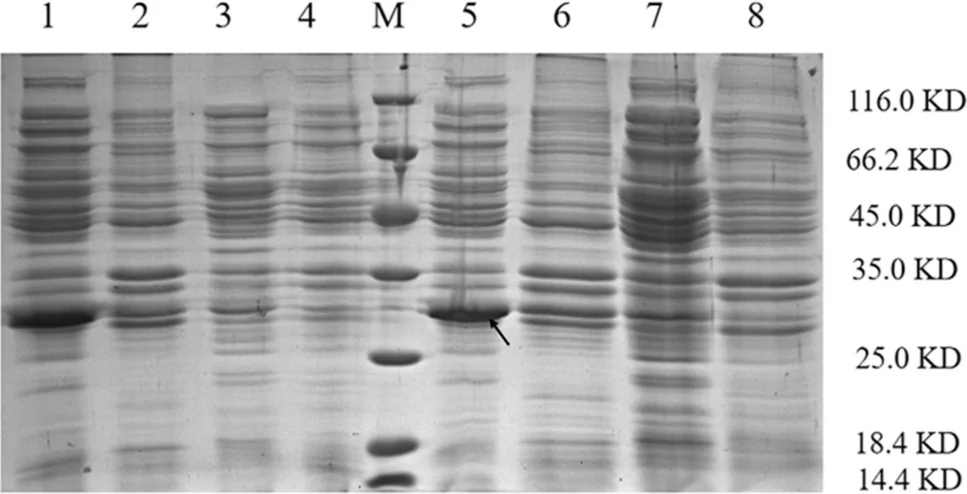
SDS-PAGE analysis of pIJ13-endolysin recombinant plasmid expression in *E. coli* JM109. Lane 1: JM109/pIJ13-lysin, supernatant (8 h); Lane 2: JM109/pIJ13-lysin, precipitates (8 h); Lane 3: JM109/pIJ13, supernatant (8 h); Lane 4: JM109/pIJ13, precipitates (8 h); M: Standard protein marker; Lane 5: JM109/pIJ13-lysin, supernatant (10 h); Lane 6: JM109/pIJ13-lysin, precipitates (10 h); Lane 7: JM109/pIJ13, supernatant (10 h); Lane8: JM109/pIJ13, precipitates (10 h). The arrow indicates the band corresponding to the endolysin protein of bacteriophage phiSASD1

To evaluate the protein expression capability of the P*SD13*, a recombinant plasmid (pIJ13-lysin) was constructed and expressed in *E. coli* JM109. Cells harboring the empty vector pIJ13 were used as a negative control. SDS-PAGE analysis revealed a distinct protein band at approximately 31 kDa in the recombinant strain, consistent with the predicted molecular weight of endolysin, whereas no corresponding band was observed in the control (Fig. 3). The target protein was predominantly detected in the soluble fraction, indicating efficient soluble expression. These results demonstrate that P*SD13* is capable of driving effective heterologous protein expression, and this improved solubility may be associated with a moderated expression level that reduces inclusion body formation commonly observed in strong expression systems.

### Structural characterization of the PSD13

To investigate the structural features of P*SD13*, transcription factor binding sites (TFBS) were analyzed using the PRODORIC database. The results indicated that P*SD13* shares high similarity with the consensus recognition sequence of the σ^70^ factor in *E. coli*. Further analysis using Softberry BPROM revealed that P*SD13* contains typical prokaryotic promoter elements, including well-defined −35 and −10 regions (Table 3). In addition, a G/C-rich spacer sequence of approximately 18 bp was identified between these two regions. These structural features suggest that P*SD13* is a σ^70^-type promoter with canonical architecture.

**Table 3:**
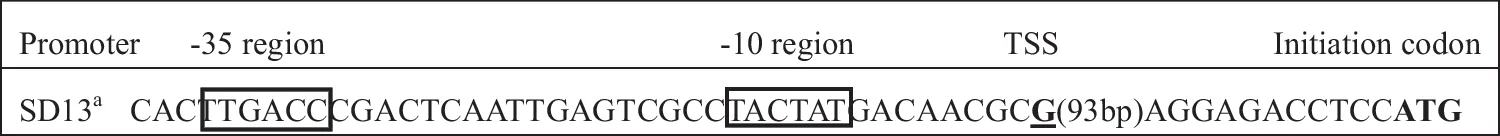
Structural analysis of the SD13 promoter fragment

^a^The sequences within the boxes represent the predicted −10 and −35 regions of the promoter. The bold and underlined base indicates the transcription start site, and the bold ATG denotes the start codon

### Determination of the transcription start site of PSD13

**Fig. 4:**
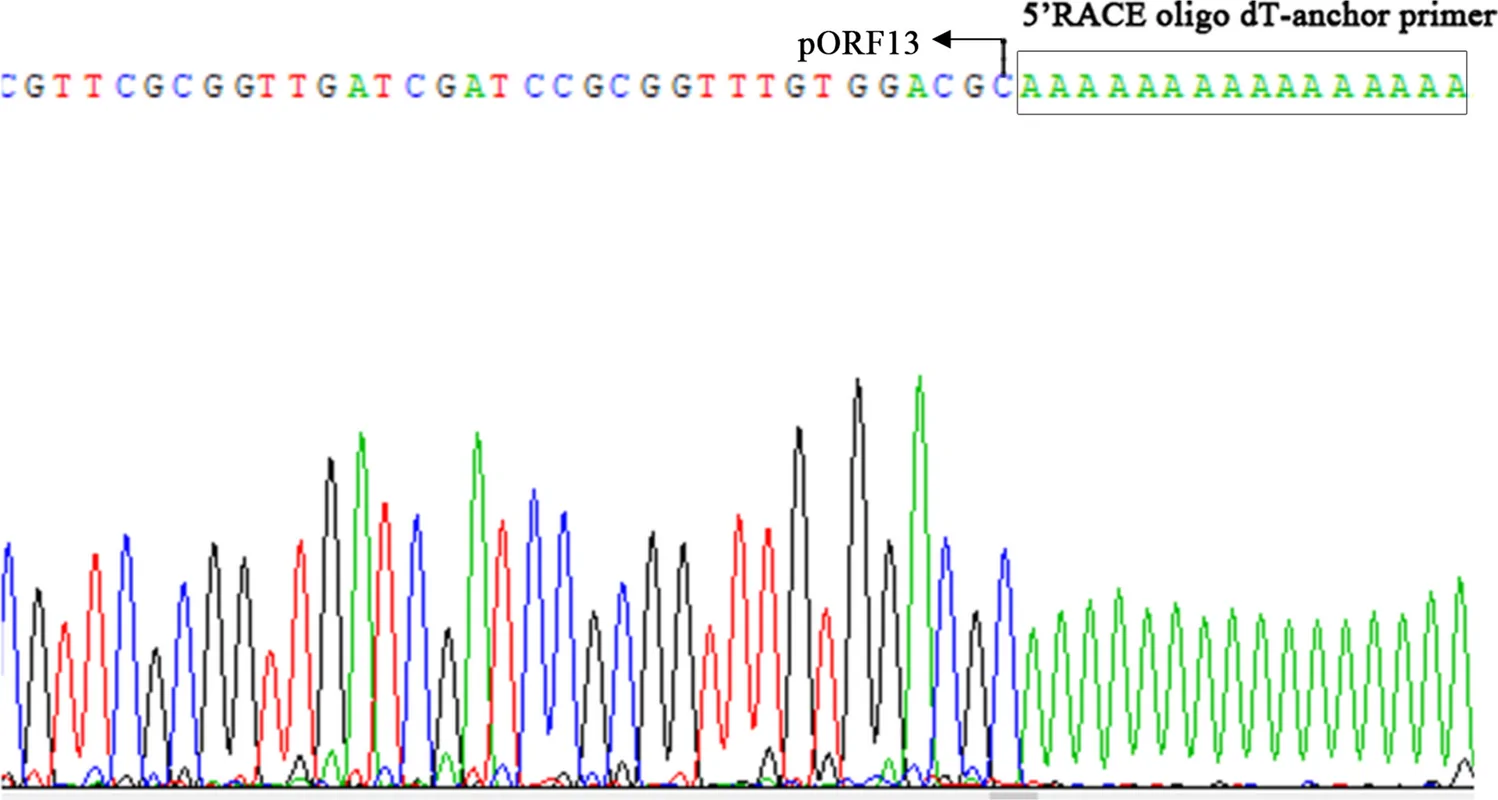
Determination of the transcription start site of the SD13 promoter by 5′ RACE. The bent arrow in the figure indicates the experimentally identified transcription start site

The transcription start site of P*SD13* was determined using 5′ RACE. The results showed that transcripts of ORF13 could be detected as early as 20 min post-infection. Sequencing of nested PCR products revealed that the TSS is located at a guanine (G) residue positioned 107 bp upstream of the start codon (Fig. 4), differing by only 1 bp from the bioinformatics prediction.

### Effects of conserved regions on PSD13 activity

**Fig. 5:**
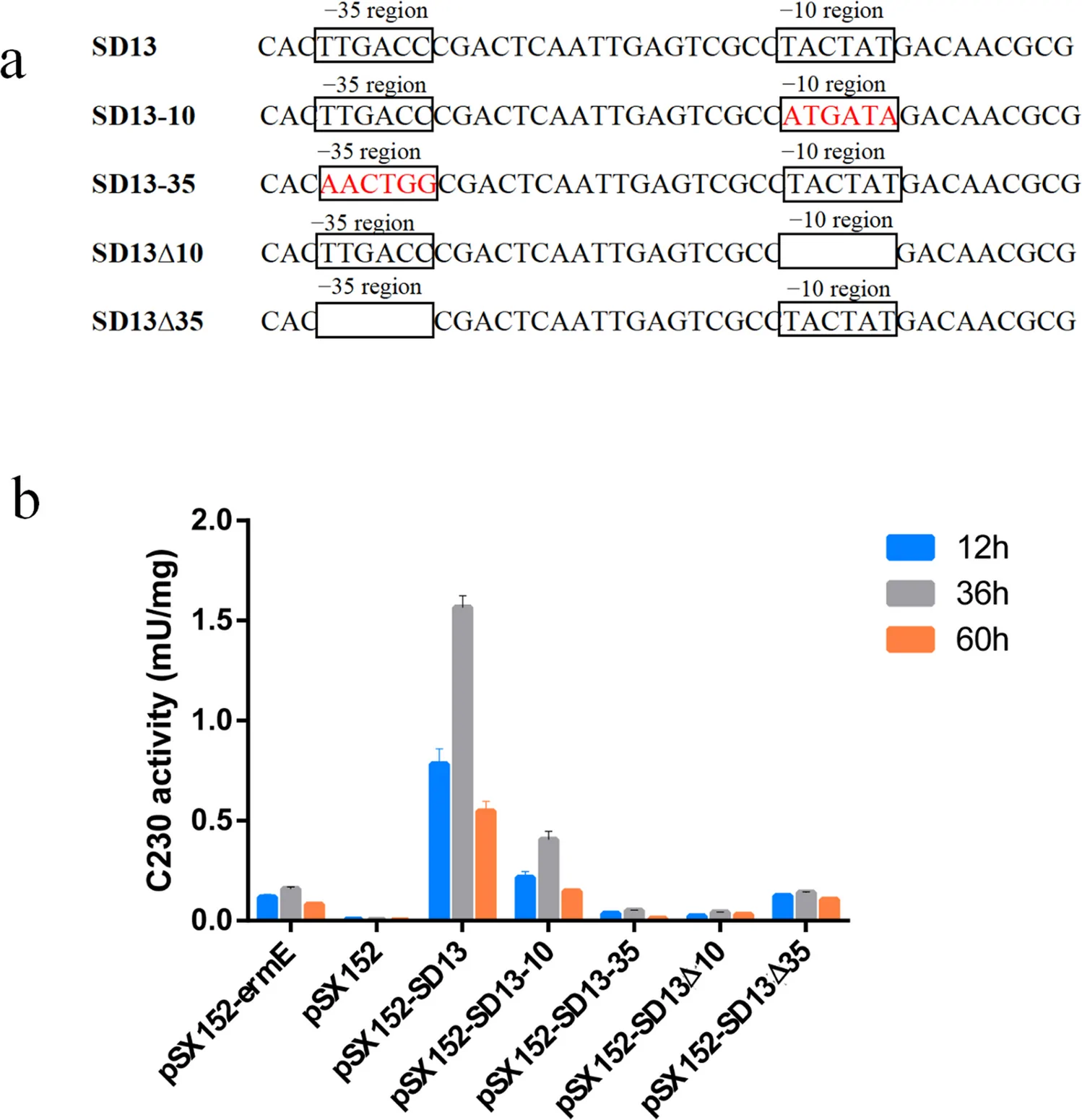
Schematic representation of mutations in the −10 and −35 regions of the SD13 promoter (**a**) and the expression activities of the corresponding chromosomally integrated mutant promoter-*xylE* cassettes in *S. lividans* TK54 (**b**). In **a**, red letters indicate that all bases in the −10 or −35 regions of each promoter were substituted with their complementary bases, while blank regions represent deletion mutations of the −10 or −35 regions

To assess the functional importance of the conserved −10 and −35 regions, site-directed mutagenesis and deletion analyses were performed. Mutation of all bases in the −10 region resulted in an approximately 3.85-fold reduction in promoter activity compared with the wild type (Fig. 5b). In contrast, mutation of the −35 region caused a much more pronounced decrease, approximately 31.34-fold (Fig. 5b). Deletion of the −10 region led to an approximately 37.67-fold reduction, whereas deletion of the −35 region resulted in an approximately 11-fold decrease in activity (Fig. 5b). These results indicate that both regions are essential for P*SD13* function, with the −35 region playing a particularly critical role in transcriptional strength.

### Identification of the core functional region of the PSD13

**Fig. 6:**
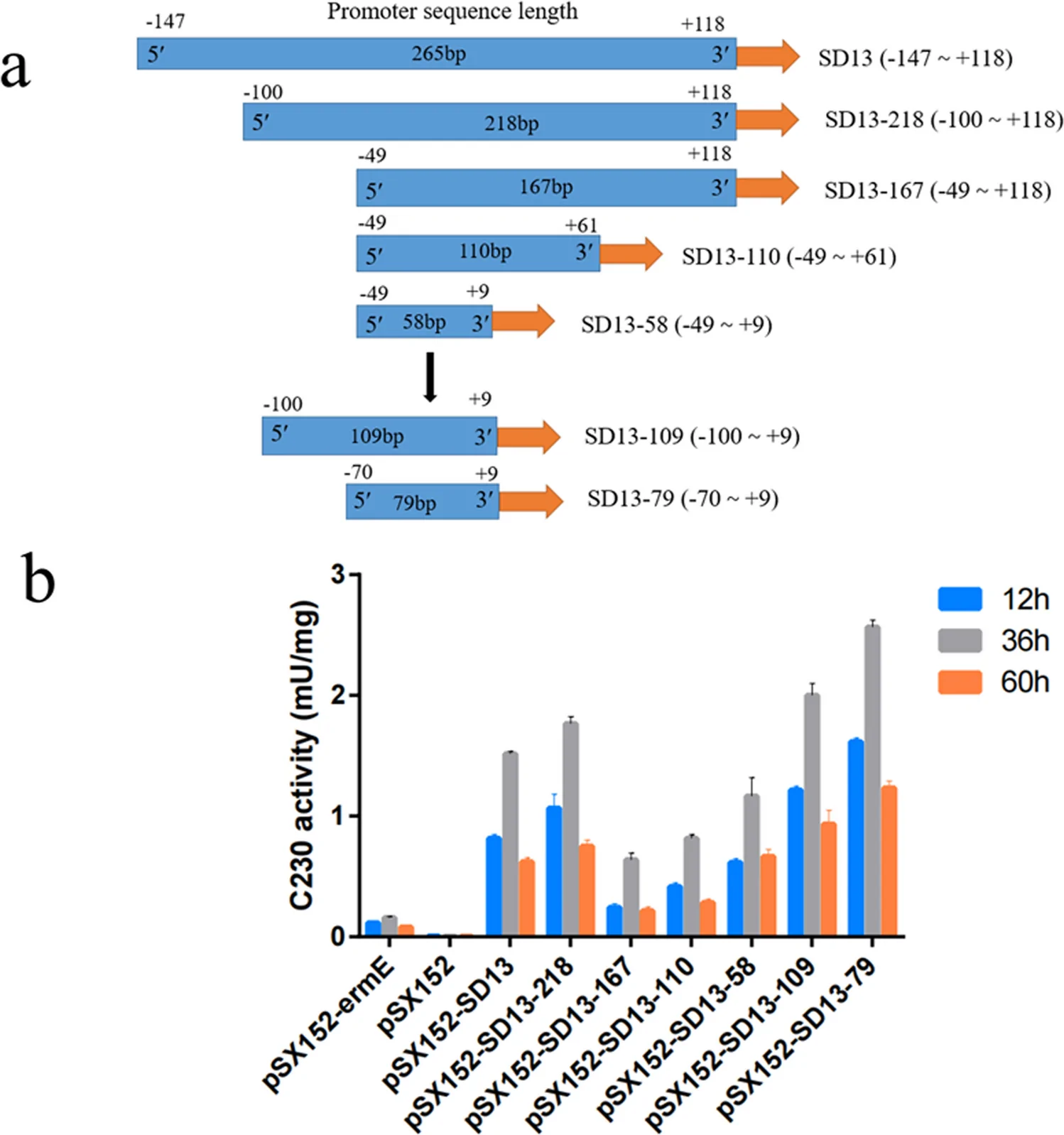
Schematic representation of 5′- and 3′-end deletion fragments of P*SD13* (**a**) and the expression activities of the corresponding chromosomally integrated promoter-*xylE* cassettes in *S. lividans* TK54 (**b**). In **a**, blue boxes indicate promoter fragments of different lengths, and the orange arrow represents the *xylE* gene. In **b**, error bars represent the standard deviation of the mean from three independent experiments

To define the minimal functional region of the P*SD13*, progressive truncation analyses were performed. The 5′-truncated fragment the *SD13*–*218* (− 100 to + 118) exhibited a slight increase in activity (1.13-fold of the wild type at 36 h) (Fig. 6b). However, further truncation to the *SD13*–*167* (− 49 to + 118) resulted in a marked reduction in promoter activity (Fig. 6b). Subsequent 3′ truncations generated the *SD13*–*110* (− 49 to + 61) and the *SD13*–*58* (− 49 to + 9) (Fig. 6a), both of which showed gradual recovery of activity (Fig. 6b). Notably, the fragment *SD13*–*79* (− 70 to + 9) exhibited the highest activity, reaching approximately 1.7-fold that of the full-length promoter at 36 h (Fig. 6b). These results indicate that the core functional region of the P*SD13* is located between − 70 to + 9 bp, and that upstream sequences may contain elements that negatively influence transcription.

## Discussion

Bacteriophages must rapidly hijack host transcriptional machinery during infection, and their genomes are therefore enriched with regulatory elements that are efficiently recognized by host RNA polymerases. In this study, we systematically screened the genome of the *Streptomyces* phage phiSASD1 and identified multiple transcriptionally active promoters, among which P*SD13* exhibited the strongest expression capability and cross-host applicability. The phiSASD1 genome, approximately 37 kb in size with 43 predicted ORFs, shares structural and organizational similarities with the well-characterized actinobacteriophage φC31 (Wang et al. 2010), supporting the concept that *Streptomyces* phages inherently harbor promoter elements compatible with host transcriptional systems. Consistent with previous reports on φC31 and φBT1, which have demonstrated strong phage-derived promoters (Howe and Smith 1996; Gregory et al. 2003). Our results provide experimental validation of multiple promoter candidates and identify one promoter exhibiting activity across both *Streptomyces* and *E. coli* hosts.

A key finding of this work is that P*SD13* showed higher transcriptional activity than the widely used constitutive promoter PermE* in *S. lividans*, reaching up to 9.79-fold under the tested conditions. However, promoter performance in *Streptomyces* is highly context-dependent, and several previously characterized constitutive promoters also display variable or condition-specific activity depending on genetic and environmental factors (Wang et al. 2013; Guo et al. 2023). Therefore, P*SD13* should not be interpreted solely as a universally superior promoter based on strength comparisons, but rather as part of an expanding and context-dependent *Streptomyces* promoter toolbox. Notably, the most prominent feature of P*SD13* is its validated cross-host functionality. In contrast to many *Streptomyces*-optimized promoters that exhibit weak or undetectable activity in *E. coli* (Baltz 2024; Nah et al. 2017), P*SD13* retained robust transcriptional output in *E. coli* JM109, where PermE* itself was inactive. This broad host compatibility may be associated with its sequence architecture, as P*SD13* contains −35/−10 motifs with high similarity to the σ^70^ consensus recognition elements in *E. coli*, which may facilitate recognition by RNA polymerase in both hosts. This observation is consistent with previous findings that conserved core promoter elements contribute to broader host range activity (Zuo et al. 2024). Meanwhile, the differential behavior of promoters such as P*SD10* and P*SD17*, which display relatively enhanced activity in *E. coli* compared with *Streptomyces*, further highlights the strong influence of host-specific transcriptional contexts on promoter performance (Horbal et al. 2013).

Another notable observation is the clear discrepancy between computational promoter predictions and experimental measurements. Several high-scoring candidates (e.g., P*SD1* and P*SD38*) exhibited no detectable activity, whereas certain low-scoring sequences such as P*SD15* showed substantial transcriptional strength. This inconsistency underscores the limitations of current promoter prediction algorithms, particularly in high-GC genomes such as *Streptomyces* and in phage-derived sequences, where regulatory architectures often deviate from canonical bacterial promoter models (Lozano Terol et al. 2021; Su et al. 2025). This bias likely stems from training datasets dominated by housekeeping promoters of model organisms, which inadequately capture phage-specific regulatory features such as temporal expression programs and alternative sigma factor recognition. These findings emphasize the necessity of experimental validation in functional promoter mining and highlight the need for improved computational frameworks tailored to actinobacteriophage regulatory systems.

Structurally, P*SD13* displays a canonical bacterial promoter architecture with well-defined −35 and −10 elements, confirmed by 5′ RACE and sequence analysis. Mutagenesis revealed that both regions are essential for activity, albeit with distinct contributions: disruption of the −35 region caused an approximately 31-fold reduction in activity, whereas mutation of the −10 region resulted in a ~ 3.85-fold decrease. Complete deletions further confirmed their indispensability, with the −35 region exerting the most pronounced effect. These results are consistent with the established roles of the −35 region in RNA polymerase recognition and initial binding, and the −10 region in DNA unwinding and open complex formation. Truncation analysis defined the minimal functional region of the P*SD13* as − 70 to + 9 bp relative to the transcription start site. Interestingly, the truncated variant the *SD13*–*79* (− 70 to + 9) exhibited even higher activity than the full-length promoter, achieving approximately 1.7-fold enhancement at 36 h. This suggests that upstream sequences (− 147 to − 71 bp) may contain inhibitory or structurally unfavorable elements that attenuate transcription. Similar regulatory effects of upstream regions, including spacer composition and DNA topology sensitivity, have been reported in other bacterial promoter systems (Klein et al. 2021). Collectively, these results define the minimal functional core of P*SD13* and provide a rational basis for future engineering to further optimize promoter strength and tunability.

From an application perspective, P*SD13* efficiently drove soluble expression of a phage-derived endolysin in *E. coli*. SDS-PAGE analysis showed that the recombinant protein was predominantly present in the soluble fraction, in contrast to the inclusion-body formation frequently observed under excessively strong T7-based expression systems. This improved solubility is likely due to moderate transcriptional output, which reduces metabolic burden and facilitates proper protein folding (Valdez-Cruz et al. 2010). Such characteristics are particularly advantageous for expressing proteins that are prone to aggregation or exhibit host toxicity. Beyond *E. coli*, the strong activity of P*SD13* in *Streptomyces* suggests broad potential for activating silent biosynthetic gene clusters and enhancing rate-limiting steps in secondary metabolite production.

Despite these promising findings, several limitations remain. First, the interaction between P*SD13* and RNA polymerase has not been directly characterized at the biochemical level, such as through electrophoretic mobility shift assays or in vitro transcription assays. Second, its performance has not been evaluated across a wider range of industrially relevant *Streptomyces* species, such as *S. coelicolor*, *S. avermitilis*, or *S. albus*, nor under fermentation conditions representative of industrial-scale production. Third, its stability and efficiency in multi-gene expression systems, as well as compatibility with inducible regulatory architectures, remain to be investigated. Future work will address these limitations, particularly by evaluating P*SD13* in the context of secondary metabolite biosynthetic gene clusters (e.g., indigoidine or actinorhodin pathways), to assess its utility in metabolic engineering and natural product biosynthesis. In addition, rational combination with optimized ribosome binding sites and transcriptional terminators may further enhance its performance and application flexibility.

In conclusion, this study demonstrates that *Streptomyces* phage genomes represent a rich reservoir of functional regulatory elements. The P*SD13* exhibits strong transcriptional activity, cross-host compatibility, and favorable protein expression characteristics, making it a promising tool for promoter engineering, metabolic pathway optimization, and synthetic biology applications. Its identification not only expands the regulatory toolbox for actinomycetes but also provides a generalizable framework for mining phage-derived promoters with broad biotechnological potential.

## Supplementary Information

Below is the link to the electronic supplementary material.

ESM 1 (DOCX 96.1 KB)

## Data Availability

The authors declared that the article and its supplementary file provide the data that supports the study’s conclusions.
