# Supplementary material for: A phage-derived promoter SD13 enables strong and cross-host gene expression in Streptomyces and Escherichia coli
Source: Appl Microbiol Biotechnol. 2026 Aug 4;110:326. doi: 10.1007/s00253-026-13981-0 (PMC13722658; doi:10.1007/s00253-026-13981-0)
Supplement: Supplementary file 1 [file 253_2026_13981_MOESM1_ESM.docx]

**Supplementary materials**

**A phage-derived promoter *SD13* enables strong and cross-host gene expression in *Streptomyces* and *Escherichia coli***

Nana Lu^1*^

^1^School of Marine and Biological Engineering, Yancheng Teachers University, Yancheng 224002, China

*Corresponding author

School of Marine and Biological Engineering, Yancheng Teachers University, Yancheng 224002, China. Email: lunn@yctu.edu.cn.

**Supplementary Fig. S1.** Original uncropped SDS-PAGE gel image corresponding to Fig. 3

**Supplementary Table S1** Bacterial strains and bacteriophage used in this study

| Strain/Phage | Relevant genotype | Source | Application |
| --- | --- | --- | --- |
| *S. avermitilis* ATCC31267 | WT | This study | Host strain for bacteriophage phiSASD1 |
| *S. lividans* TK54 | WT | This study | *Streptomyces* expression host |
| *E. coli* JM109 | *recA1, endA1, gyrA*96*, thi-*1*, hsdR*17*, supE*44*, relA1,* Δ*(lac-proAB)/*F*’* [*traD*36*, proAB+, lacIq, lacZΔM15*] | Takara | Plasmid construction, propagation, promoter activity assay, heterologous protein expression |
| Bacteriophage phiSASD1 | Temperate siphovirus; dsDNA genome (37,068 bp); infects *S. avermitilis*; GenBank accession no. GQ379227 | This study | Promoter mining |
| *E.coli* ET12567/pUZ8002 | dam, dcm, hsdM, carrying helper plasmid pUZ8002 | This study | Intergeneric conjugation donor for transfer of recombinant plasmids into *Streptomyces* |

**Supplementary Table S2** Plasmids used in this study

| Plasmid | Backbone | Marker | Insert | Purpose |
| --- | --- | --- | --- | --- |
| pSET152 | φC31 integrative shuttle vector | aprR | *attP/in*t | Construction of promoter probe vector |
| pIJ4083 | Reporter plasmid carrying *xylE* | tsrR | *xylE* | Source of the *xylE* reporter gene |
| pSX152 | Promoterless *xylE* reporter vector derived from pSET152 | aprR | *xylE* | Promoter activity assay |
| pSX152-PermE* | pSX152 carrying PermE* | aprR | P*ermE** | Positive control |
| pSX152-SDX | pSX152 carrying individual phiSASD1 promoter fragments | aprR | Individual promoter fragments | Promoter activity characterization |
| pIJ13 | pIJ8630 derivative carrying the SD13 promoter | tsrR | SD13 promoter | Heterologous protein expression |
| pIJ13-lysin | pIJ13 carrying the endolysin coding sequence | tsrR | phiSASD1 endolysin gene | Expression of recombinant phage endolysin |

**Supplementary Table S3** Primers used in this study

| Primer name | Sequence (5′ → 3′) | Purpose | Application |
| --- | --- | --- | --- |
| SD1-F | AGGGTACCGGCACGCTAACCTGATCC | SD1 promoter region amplification | Promoter amplification |
| SD1-R | AAGGTACCGGTGCTGATGGGATTGG | SD1 promoter region | Promoter amplification |
| SD3-F | AAGGTACCGTGCCCACTGAAGAGGAG | SD3 promoter region | Promoter amplification |
| SD3-R | AAGGTACCGATCGGTACAACGTCCATC | SD3 promoter region | Promoter amplification |
| SD5-F | TGGTACCCTGACCCGTTAACTCTCC | SD5 promoter region | Promoter amplification |
| SD5-R | AGGTACCCATCGTGAACCCCTCTC | SD5 promoter region | Promoter amplification |
| SD8-F | TCGGTACCGAAGACGAACGTATTGAC | SD8 promoter region | Promoter amplification |
| SD8-R | ATGGTACCTGTCCCCGATGCTGTAG | SD8 promoter region | Promoter amplification |
| SD10-F | AAGGTACCTTCACTACACGGCCCAG | SD10 promoter region | Promoter amplification |
| SD10-R | AGGTACCGCCTTCGAAGGGTTAGC | SD10 promoter region | Promoter amplification |
| SD11-F | AGGTACCCCGCCTGTCTGACGTCT | SD11 promoter region | Promoter amplification |
| SD11-R | AGGTACCCAGAATCGCGGACATCTC | SD11 promoter region | Promoter amplification |
| SD12-F | AAGGTACCATCTTCGGGGAGTAGCG | SD12 promoter region | Promoter amplification |
| SD12-R | AGGTACCCTGAAGTGGCGCATGTT | SD12 promoter region | Promoter amplification |
| SD13-F | TAGGTACCTGTACGGCGCTGATTACT | SD13 promoter region | Promoter amplification |
| SD13-R | TAGGTACCGTGACAGACATGGAGGT | SD13 promoter region | Promoter amplification |
| SD15-F | CTTCTGGCTGTCGTATGC | SD15 promoter region | Promoter amplification |
| SD15-R | CTGATCGTCAACGGTCTG | SD15 promoter region | Promoter amplification |
| SD17-F | AGGTACCGGTGAGGCGCATGGAGG | SD17 promoter region | Promoter amplification |
| SD17-R | ACGGTACCCGGGAGGTCAAGACGTTC | SD17 promoter region | Promoter amplification |
| SD18-F | TCTACAAGACGGCGAAGT | SD18 promoter region | Promoter amplification |
| SD18-R | GTGATGAGCGACAGGAAG | SD18 promoter region | Promoter amplification |
| SD19-F | AGGTACCTCTGGGCGATCACTGGT | SD19 promoter region | Promoter amplification |
| SD19-R | AGGTACCCGTCCACGAGCTCAAATT | SD19 promoter region | Promoter amplification |
| SD21-F | AAGGTACCGTTCAAAGACCTGGTGTG | SD21 promoter region | Promoter amplification |
| SD21-R | ATGGTACCAACCCTGGGAGTCTGTC | SD21 promoter region | Promoter amplification |
| SD38-F | AGGTACCGAAAACCAGGTCGTGAT | SD38 promoter region | Promoter amplification |
| SD38-R | TCGGTACCCGTGTAGCGGAATATAA | SD38 promoter region | Promoter amplification |
| SD40-F | AAGGTACCCCTGTCCATCTCCAGC | SD40 promoter region | Promoter amplification |
| SD40-R | AGGTACCTGTCCATCGCACCACC | SD40 promoter region | Promoter amplification |
| SD13-218-F | TAGGTACCCTGAACGCAACCGAAGC | 5′-truncated of SD13 promoter | Construction of 5′-deletion variants |
| SD13-218-R | TAGGTACCGTGACAGACATGGAGGT | 5′-truncated of SD13 promoter | Construction of 5′-deletion variants |
| SD13-167-F | TAGGTACCTCAGCCGCCTCACTTGAC | 5′-truncated of SD13 promoter | Construction of 5′-deletion variants |
| SD13-167-R | TAGGTACCGTGACAGACATGGAGGT | 5′-truncated of SD13 promoter | Construction of 5′-deletion variants |
| SD13-110-F | TAGGTACCTCAGCCGCCTCACTTGAC | 3′-truncated of SD13 promoter | Construction of 3′-deletion variants |
| SD13-110-R | TAGGTACCATGAAGGTGGAAGGAATG | 3′-truncated of SD13 promoter | Construction of 3′-deletion variants |
| SD13-58-F | TAGGTACCTCAGCCGCCTCACTTGAC | 3′-truncated of SD13 promoter | Construction of 3′-deletion variants |
| SD13-58-R | TAGGTACCTGTGGACGCGTTGTCAT | 3′-truncated of SD13 promoter | Construction of 3′-deletion variants |
| SD13-SP1 | GCAGACTCGGCAAGGTTC | SP1 for reverse transcription | 5′ RACE (cDNA synthesis) |
| SD13-SP2 | TTCACCATCTGGGCGAAC | Nested SP2 for PCR1 | 5′ RACE (primary PCR) |
| SD13-SP3 | GTCGCTGTCGTCGTCCGT | Nested SP3 for PCR2 | 5′ RACE (nested PCR) |
| SD13-SP4 | CAACTCGCGCTCCACGTA | Nested SP4 for PCR3 | 5′ RACE (secondary nested PCR) |
| endolysin-F | CGTCTAGAATGGACAGCCCTTCGCGT | Amplification of endolysin gene | Cloning of endolysin into expression vector under SD13 promoter |
| endolysin-R | AGGATCCTCAGGCGGTCACGGTCC | Amplification of endolysin gene | Cloning of endolysin into expression vector under SD13 promoter |

**Supplementary Table S4** Nucleotide sequences of experimentally validated active promoter fragments from bacteriophage phiSASD1

| Promoter | Genomic location | Length (bp) | Nucleotide sequence (5′→3′) |
| --- | --- | --- | --- |
| P*SD10* | 24813–25070 | 258 | TTCACTACACGGCCCAGGCCCCGGAGCCGGCATGACCGCCTGGTCCGTACTGGTCCGCGTCCTGTCCCTGGTCGCTGTCCTCGCCGCTCTGGCCTGGGCCCTGGACCTTTTCCCCTGGCAGAGGAGGCGTTAACGATGACTTCCCCCT  TCACTGAACTGGCCGCTGTCGCTGGCGGCATCGTCCTGGCCGCGGTCATCGTCGCCGTGGCCCACCTGATCCACGTTCTG GGCGGTCGCC GTGGCTAACC CTTCGAAGGC |
| P*SD12* | 28111–28297 | 187 | ATCTTCGGGGAGTAGCGGGACCGGGGGACATCCTCTCCGGACAGTCGTGAAGCCGCTGATCCCCCGCACTTCCGACCGCC TGGCACGCACCCAGGCTCCG TGTGATCACC CAGGGCCGGACCTCTTGACGGGGGTCCGGCCTTCGCCCTGTCCAAACACGGAGAGGGGACAACATGCGCCACTTCAG |
| P*SD13* | 30121–30385 | 265 | TGTACGGCGCTGATTACTGATCGGTCCTGACCGACTCGTGTTCGGCCCTGAACGCAACCGAAGCCACTTGTGTTCAGGGCCTTCACCAGATCGTTACATCAGCCGCCTCACTTGACCCGACTCAATTGAGTCGCCTACTATGACAACGCGTCCACAAACCGCGGATCGATCAACCGCGAACGGAACGACGCATTCCTTCCACCTTCATGCCCCCTGGCTTCGCCATGCCCGGGGGCCTCCCAGAGGAGACCTCCATGTCT GTCAC |
| P*SD15* | 32181–32330 | 150 | TGGACGACGAAGGCGAGTGAACGGCTTGACTCGACTCAGTTGAGTCGGCTACTTTCGGCATTCCAGCCGCCCCTACTGAGGCACCTACCTGAGAGGCGTAGACCAATGACCGCCATCCGCACTCGCCTGACCGTGGCCGCCGGCCTGGTC |
| P*SD17* | 33791-34450 | 660 | CCAAGGTGAGGCGCATGGAGGCCAATGGGGTGGACGTAGAGTCCTGGCTGGCGCCGATGTAGGCGGCCCAGGGGCCGACAGCGGGCGAGTTGTTTCTATATTTTTGTTTCGTCCCACTATCTAAAATAACTCTCTACCAGCTCCGCTCTTTGGGCCTTAACCGTTGAACTGGGACATTTCTCGGCTTCCGCAACAGCCCACATCAGGGGACCTCCTCCCCGGTCAGGTGACGTACCCGCCCGCCCGGTATGAGGAGGTCCCTTGACCGTCTATGAAACCCATTGCGCGCATTGCGGCGAAGGCTTGACCGGCCGTCAGCGTCGGACGTGCTCCCCGCGGTGCCGTAAGGCGATCCAGCGCACGTCACCTGCCCTCCGCACGTGCAAGCTGTGTGCCCAGCCCTTCAAGCCGTCCGGCCCTGGCCGCCGCACCGTCTGCCCGTACGACGACGCCGACGACTTCTGTCAGGGCCTCCAGGACGACCAGGAAGACGCACTGGCCGCCCAGGCAGCCGCGCGCGAAGAGGCCACGTGCGAGGGATGCCGCCAGCCGCTGGCTTACGCCGGCCGTGGCCGCCCGCCTCGCTTCTGTGGTCCCGCTTGCAAGACGCGCACCTACCGGAAGGCGGCCTCCGCGTGAACGTCTTGACCTCCCGTACCG |
| P*SD18* | 34997-35173 | 176 | GCTCTTCGACGTTCACGAACGCCTGACGGACGCGGAGCTGGCTGCCCGCCAGGCCGGCGGAGGCGACTCAAGTGAAGCGGCTTGACTCGACTCAATTGAGCCGGCTAAATTGGAGCCCTCGCCAGGGTGGCGGGGAGGAGAGGACCCAGACATGACCCGCACAGAGATCTACAAGA |
| P*SD40* | 16459-16602 | 144 | TCCCCCTCTTCCCCCTTCTCTGGCCCCCGTCCTCGGCATCGCTGCCCAGGCGGGGGCCCTCGCATGAAAGGACCCTCATGCCCACGATATGGATTCCTGGCGCGGAGCGTCTCGGCGCAGGCTCGATCGGTGGTGCGATGGA CA |
